# Supplementary material for: Causal Relationship between Aging and Anorexia Nervosa: A White-Matter-Microstructure-Mediated Mendelian Randomization Analysis
Source: Biomedicines. 2024 Aug 16;12(8):1874. doi: 10.3390/biomedicines12081874 (PMC11351342; doi:10.3390/biomedicines12081874)
Supplement: Supplementary file 1 [file biomedicines-12-01874-s001.zip › Supplementary Figure/Figure S4/Singe SNP plot of MR analysis for TL to ubm-b-1588.pdf]

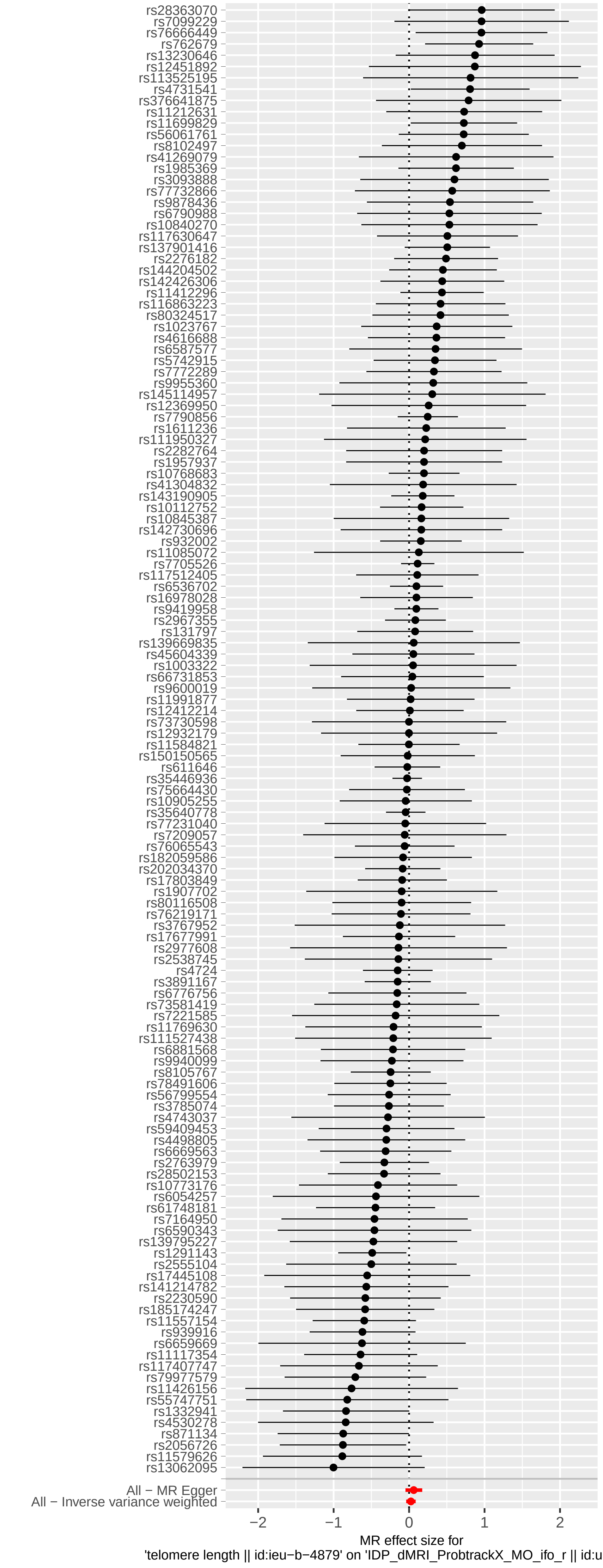
rs28363070
rs7099229
rs76666449
rs762679
rs13230646
rs12451892
rs113525195
rs4731541
rs376641875
rs11212631
rs11699829
rs56061761
rs8102497
rs41269079
rs1985369
rs3093888
rs77732866
rs9878436
rs6790988
rs10840270
rs117630647
rs137901416
rs2276182
rs144204502
rs142426306
rs11412296
rs116863223
rs80324517
rs1023767
rs4616688
rs6587577
rs5742915
rs7772289
rs9955360
rs145114957
rs12369950
rs7790856
rs1611236
rs111950327
rs2282764
rs1957937
rs10768683
rs41304832
rs143190905
rs10112752
rs10845387
rs142730696
rs932002
rs11085072
rs7705526
rs117512405
rs6536702
rs16978028
rs9419958
rs2967355
rs131797
rs139669835
rs45604339
rs1003322
rs66731853
rs9600019
rs11991877
rs12412214
rs73730598
rs12932179
rs11584821
rs150150565
rs611646
rs35446936
rs75664430
rs10905255
rs35640778
rs77231040
rs7209057
rs76065543
rs182059586
rs202034370
rs17803849
rs1907702
rs80116508
rs76219171
rs3767952
rs17677991
rs2977608
rs2538745
rs4724
rs3891167
rs6776756
rs73581419
rs7221585
rs11769630
rs111527438
rs6881568
rs9940099
rs8105767
rs78491606
rs56799554
rs3785074
rs4743037
rs59409453
rs4498805
rs6669563
rs2763979
rs28502153
rs10773176
rs6054257
rs61748181
rs7164950
rs6590343
rs139795227
rs1291143
rs2555104
rs17445108
rs141214782
rs2230590
rs185174247
rs11557154
rs939916
rs6659669
rs11117354
rs117407747
rs79977579
rs11426156
rs55747751
rs1332941
rs4530278
rs871134
rs2056726
rs11579626
rs13062095
All − MR Egger
All − Inverse variance weighted
−2
−1
0
1
2
MR effect size for
'telomere length || id:ieu−b−4879' on 'IDP_dMRI_ProbtrackX_MO_ifo_r || id:ub
